# 

**Published:** 1887-10-15

**Authors:** 


PRICE ONE PENNY.

No. 55, Vol. III.

October 15th, 1887.

[Registered at the General Post Office as a Newspaper.]

Per Annum, 6s. 6d., post free.

Monthly Parts, 6d.; post free, 7½d.

